# Water Quality Monitoring for Lake Constance with a Physically Based Algorithm for MERIS Data

**DOI:** 10.3390/s8084582

**Published:** 2008-08-05

**Authors:** Daniel Odermatt, Thomas Heege, Jens Nieke, Mathias Kneubühler, Klaus Itten

**Affiliations:** 1 Remote Sensing Laboratories (RSL), UZH, Winterthurerstr. 190, CH-8050 Zurich, Switzerland; 2 EOMAP GmbH & Co. KG, Sonderflughafen Oberpfaffenhofen, D-82205 Gilching, Germany; 3 ESA/ESTEC, 2000AG Noordwijk, The Netherlands

**Keywords:** remote sensing, inland water, chlorophyll, monitoring, operationalization

## Abstract

A physically based algorithm is used for automatic processing of MERIS level 1B full resolution data. The algorithm is originally used with input variables for optimization with different sensors (i.e. channel recalibration and weighting), aquatic regions (i.e. specific inherent optical properties) or atmospheric conditions (i.e. aerosol models). For operational use, however, a lake-specific parameterization is required, representing an approximation of the spatio-temporal variation in atmospheric and hydrooptic conditions, and accounting for sensor properties. The algorithm performs atmospheric correction with a LUT for at-sensor radiance, and a downhill simplex inversion of chl-a, sm and y from subsurface irradiance reflectance. These outputs are enhanced by a selective filter, which makes use of the retrieval residuals. Regular chl-a sampling measurements by the Lake's protection authority coinciding with MERIS acquisitions were used for parameterization, training and validation.

## 1. Introduction

Monitoring of water quality in lakes is an integral part of water resource management. It ensures the sustainable use of water and allows tracking the effects of anthropogenic influences. Water quality monitoring of the large fluviglacial Swiss lakes was established in the 1950s and 1960s. A broad range of water quality parameters is sampled at decent temporal resolutions, but very limited in the spatial dimension. In the early 1990s, analytical methods applied to high spectral resolution airborne scanner data were found to bear the potential to overcome these limitations. But neither did these studies lead to operational algorithms, nor was an adequate space borne sensor for monitoring purposes available at the time [1]. The latest generation of medium resolution Earth observation sensors (i.e. Moderate Resolution Imaging Spectroradiometer MODIS, Medium Resolution Imaging Spectrometer MERIS) provide a nominal revisit time of 2-3 days at mid latitudes and could therefore be an effective means to provide spatial measurements. A recent MERIS algorithm based on neural networks [2] improved the applicability of remote sensing data to optically complex waters (i.e. case II), and validation experiments confirmed the potential of satellite remote sensing for inland water quality monitoring, but at the same time revealed shortcomings concerning especially atmospheric correction [3].

MIP (Modular Inversion and Processing System) is an alternative algorithm based on the minimization of the difference between satellite measured and modeled spectra. It was developed for use with airborne sensors, where changing image acquisition conditions require higher flexibility [4, 5]. MIP was originally designed for Lake Constance, but has been used for different industrial and research applications in several marine (e.g. coast of Western Australia, Indonesia) and limnic (e.g. Lake Sevan/Armenia, Mekong/Vietnam, Lake Starnberg and Lake Waging-Taching/Germany) environments. The aim of this work is to make MIP applicable for automatic processing by optimizing a single, lake specific parameterisation for MERIS data of Lake Constance.

Lake Constance is the second largest lake in Western Europe, covering an area of 535 km^2^ shared by Austria, Germany and Switzerland. It is located at 395 m a.s.l., its average and maximum depth are 101 m and 253 m, respectively. 15% of its area is shallow water of less than 10 m depth. The Alpenrhein River is its main feeder, accounting for 62% of the total inflow. Originally oligotrophic, the eutrophication of Lake Constance reached a peak in the late 1970s, mainly due to nutrient influxes, followed by 20 years of steady reoligotrophication [6, 7]. Bi-weekly water quality monitoring measurements are carried out by IfS, on behalf of IGKB. Total phosphorous concentrations are still decreasing, e. g. from 10 mg/m^3^ in spring 2003 to 8 mg/m^3^ in spring 2005. Highest chlorophyll-a concentrations are reached during spring blooms, with a maximum of 11.8 μg/l in the top 10 m layer on 19 March 2002, but possibly higher concentrations at the water surface. Apart from 2002, spring blooms occurred earlier and at a smaller extent in recent years. In 2005, it started in late March and reached its peak in mid April, two weeks earlier than in 2003 [8]. Other than that, seasonal variations of chlorophyll-a concentrations are between 1 μg/l in winter and 3-5 μg/l in summer and autumn [7].

## 2. Data

### 2.1. Satellite data

51 MERIS level 1B full resolution datasets [9] of Lake Constance with coinciding IGKB water quality measurements are used in total. Both 2241 square pixel scenes and 1153 square pixel quarter scenes (“imagettes”) are processed. MERIS data consist of 15 spectral channels as described in Table 1, at a ground resolution of about 300 m, and metadata, including geolocation, geometry and quality flag layers. Smile correction was not applied.

**Figure 1.**
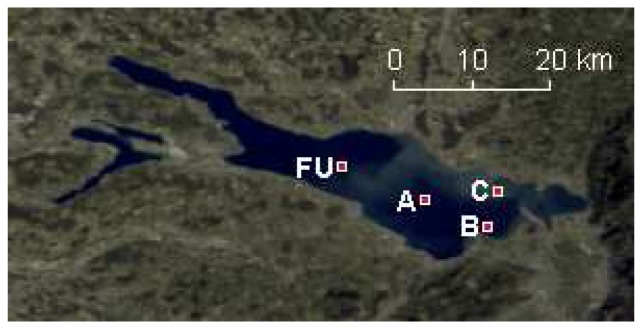
MERIS true color composite of Lake Constance, acquired 20 April 2007. Fischbach-Uttwil (FU) and the measurement sites A to C are located in the main basin called Obersee, with the finger-shaped Lake Überlingen in the top left corner of the image and the separated Untersee below. Geometric correction was not applied; the scale is averaged for the lake surface.

In pre processing, MERIS geolocation metadata is searched for the center coordinates of Lake Constance. A 501 to 301 pixels subset of all channels is extracted where these coordinates are found (Figure 1). The clippings include scaled radiances of all channels and are saved in BIL (Band Interleaved by Line) format. Meta data such as observation date, time and geometry, geolocation data and pixel quality flags are added for use in MIP modules and post processing. Georeferencing is not performed.

Among the total 51 images processed, a total of 18 images could not be further used in this study (Table 2). The data were excluded due to 3 different reasons:

(1) Sun glint occurs for certain observation geometries and rough water surfaces (i.e. high wind speed). It increases reflected NIR radiance, and thus causes errors in atmospheric correction. MERIS sun glint warning flags aren't set for inland waters, and wind speed metadata is not applicable over land. However, in the summer half-year, even 1 m/s wind speed on Lake Constance causes 1% sun glitter reflection at 20° eastward viewing zenith angle [10]. Eight erroneously processed images acquired at more than 20° eastward zenith in the summer half-year were therefore considered to be affected by sun glint.
(2) Cirrus clouds or contrails are visible in 6 images, although they are not identified by the MERIS bright pixel flags.
(3) MIP's atmospheric correction module is unable to process 4 images, in which aerosol optical thicknesses (AOT) is overestimated and reflectances in channels 1, 2, 6, 7 and 8 become zero [11].

### 2.2. Field campaign data

**Figure 2.**
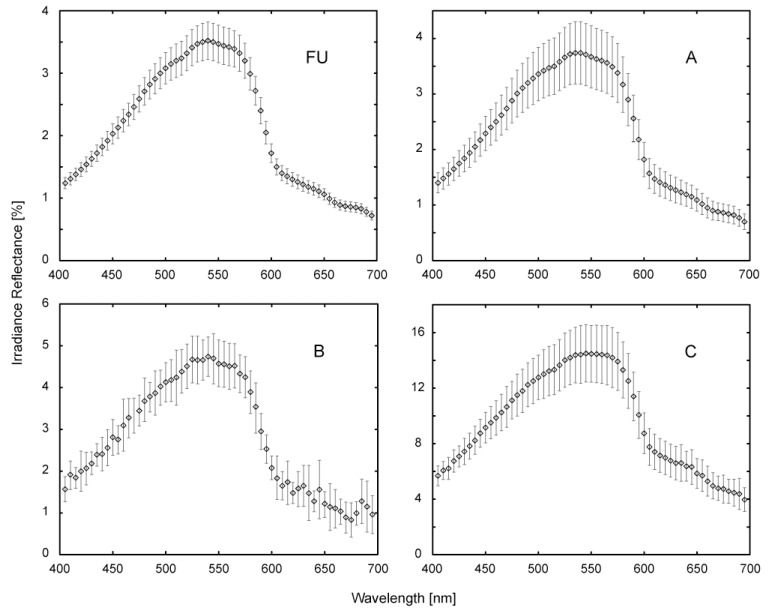
RAMSES data acquired in the sites FU and A-C (Figure 1) at a depth of 20 cm, on 20 April 2007.

On 20 April 2007, up- and downwelling irradiances E_u_ and E_d_, were measured in situ during MERIS overpass, R^-^ was calculated through Equation 1. The measurements with two RAMSES AAC instruments [12] onboard a research vessel of IfS were taken in the 4 sites depicted in Figure 1. Each dataset is an average of more than 20 5 s sampling intervals. The data is spectrally binned to 70 channels between 350 and 700 nm, at uniform intervals of 5 nm. Measurements were taken about 20 cm below the water surface and at 1 m depth. The relatively higher variations in the water column above the instrument during the 20 cm measurements caused generally smaller standard deviations than the low signal level at 1 m depth, the 20 cm data was thus preferred for further analysis (Figure 2). However, some instrument noise persists, even after manual removal of outliers, especially at 600-700 nm in the data of site B.

(1) $$R^{-}=E_{u}^{-}/E_{d}^{-}$$

Reference measurements of constituents are taken from water samples. Suspended matter (sm) is measured as sum of organic and inorganic matter not passing a 1 μm glass fiber filter [5]. Gelbstoff (y) is filtered through a 0.2 μm filter and measured in a laboratory spectrometer [13]. However, the results are strongly inconsistent with one another, we can therefore only compare the y concentrations of MERIS and RAMSES inversion. Chlorophyll-a (chl-a) was measured with a fluorometer probe, which is cross-calibrated with HPLC (High Performance Liquid Chromatography) measurements by IfS.

### 2.3. Water quality monitoring data

In situ chl-a measurements carried out by IfS as part of the water quality monitoring by IGKB are used for training and validation of MERIS processing results. The data were sampled at the site Fischbach-Uttwil (FU, Figure 1, 47.62N / 9.37E), in approximately bi-weekly intervals. FU is located in the lake's deepest area and was chosen for comparison with satellite data because the disturbance by adjacency effects occurring in MERIS data is minimal in the pelagic [11]. The method used for chl-a determination is HPLC [14, 15]. 103 in situ measurements are available for the investigation period 2003-2006. Concurring measurements are available for 47 MERIS images; 4 dates in 2006 were interpolated from consecutive IGKB measurements with only small variation.

The chl-a concentrations measured by IfS represent an integral of the top 20 m layer, whereas the estimate from MERIS data represents only the top layer from which the signal originates. In Lake Constance, the top 2 (blue, red) to 8 m (green light) account for 90% of the reflected radiance, when the water is very clear. But in turbid waters, the same part of reflected radiance may be from only 1 and 2 m, respectively [5]. This means that vertical variations in water constituent concentrations, which are included in the 20 m column samples, will not be represented by estimates from remote sensing. However, the analysis of more than 350 profiles for both chl-a and sm in Lake Überlingen revealed a strong vertical correlation between the top layer at 0.5-1.5 m and the layers below [5, 16].

## 3. Methods

### 3.1. Algorithm description

The MERIS level 1B FR data are processed with two MIP modules [4, 5]. The first MIP module performs image based aerosol retrieval and atmospheric correction on at-sensor radiance data. It uses a look up table (LUT), which was simulated with a coupled, plane-parallel atmosphere-water model and the finite element method [17]. The module relates at-sensor radiances L_s_ to AOT of either continental, maritime or rural aerosol type, observation geometry, wavelength and the subsurface radiance reflectance R_L_^-^, which is mainly due to backscattering on suspended matter (sm) at large wavelengths. The resulting AOT map is used to retrieve the angularly dependent subsurface radiance reflectance R_L_^-^ for channels 1-8 from the same LUT. Another LUT is used to account for the directionality of the underwater light field, thus to convert R_L_^-^ to the angularly independent subsurface irradiance reflectances R^-^. It consists of Q-factors for varying wavelengths, observation geometries and water constituent concentrations, and is applied to R_L_^-^ according to Equation 2.

(2) $$R^{-}=R_{L}^{-}(\Delta \phi ,\theta_{\text{obs},}\theta_{\text{sun}})\pi /Q(\Delta \phi ,\theta_{\text{obs}},\theta_{\text{sun}})$$

The inherent optical properties (IOP) of water are related to R^-^ through Equation 3 [18], where *f* is parameterized as a function of μ [19], and μ is calculated for z = 0 m as a function of a, b, and the mean cosine of the incident light field [20].

(3) $$R^{-}=f\;b_{b}/(a+b_{b})$$

The coefficients x_i_ for absorption (x=a), scattering (x=b) and backscattering (x=b_b_) of pure water (i=w), chlorophyll-a (i=chl-a), suspended matter (i=sm), and gelbstoff absorption (i=y) are calculated by Equation 4, whereas a_sm_, b_chl-a_ and b_y_ can be neglected for Lake Constance [4, 5].

(4) $$x=x_{w}+x_{\text{chl}-a}\text{chl}-a+x_{sm}+x_{y}y$$

**Figure 3.**
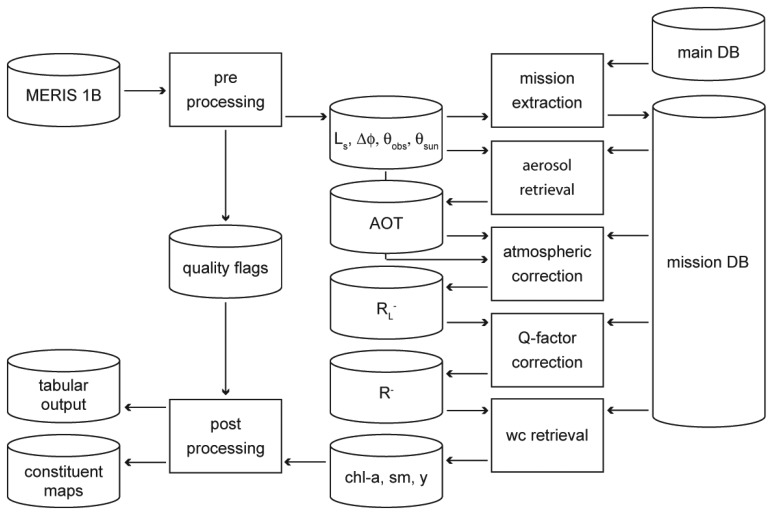
Flow chart of the automatic data processing chain. The mission DB contains the LUTs for atmospheric and Q-factor correction, for the data specifications defined in the mission extraction. The tabular output contains concentration and retrieval quality parameters for FU and lake means.

The inversion of subsurface irradiance reflectance R^-^ to the coefficients x_i_ is accomplished by another MIP module. It adjusts modeled and input image spectra after atmospheric correction by means of a downhill simplex algorithm [21]. The algorithm starts with a set of initial concentrations. The spectrum modeled for these concentrations is linearly scaled to fit the input spectrum, leading to a first guess of concentrations, which is then optimized by two iterations of Q-factor correction and water constituent retrieval. The full processing scheme is illustrated in Figure 3.

**Figure 4.**
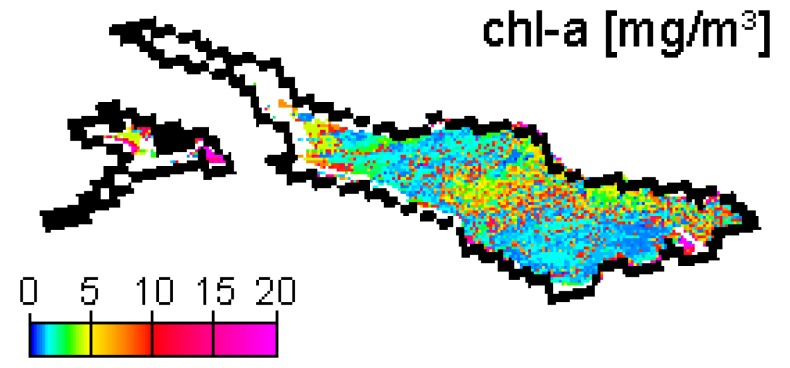
Chl-a map for 20 April 2007, prior to filtering.

**Figure 5.**
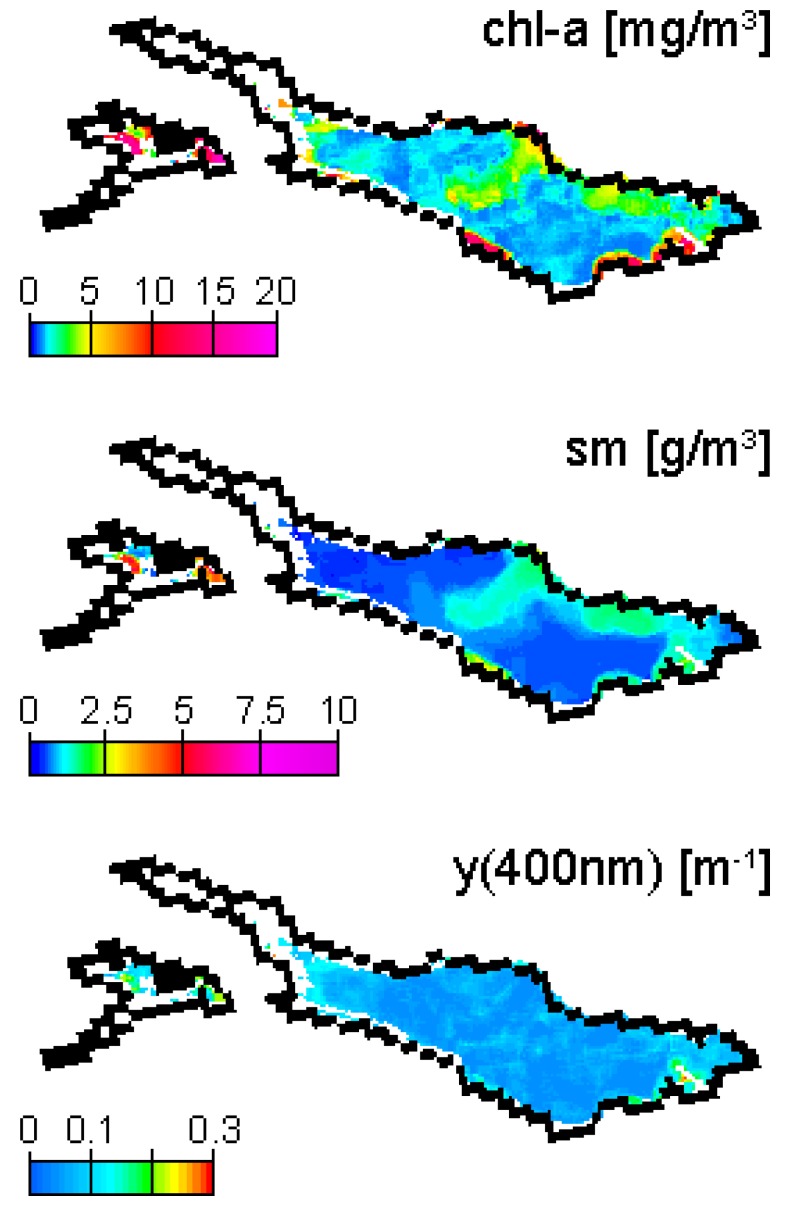
Chl-a, sm and y map for 20 April 2007, after application of the selective filter.

MIP generates maps of chl-a and sm concentration, y absorption (400 nm) and AOT (550 nm). Furthermore, residuals of image and model spectra fits are calculated as a retrieval quality indicator. Occasional over- and underestimation of AOT by the atmospheric correction module may cause zero reflectances in red bands or a shift of the reflectance peak towards the blue bands, respectively. This may force the constituent retrieval algorithm to approximate irregular spectral shapes, leading to high variations between neighboring pixels, and in places the algorithm reaches its threshold of 20 mg/m^3^ (Figure 4). Such aberrations can be reduced by a low pass filter on input imagery [22], as SNR in MERIS channels 1-8 of reduced resolution (RR) data decreases from about 1:1100 to 1:500, but is very close to 4 times lower in FR data [23, personal communication]. In order to use the retrieval residual as an indicator of whether the atmospherically corrected R^-^ are valid water spectra reproducible by the model, we combined such spatial smoothing with a selective filter, which replaces each output concentration pixel by the average of the concentrations of the 3 pixels fitted at the lowest residual within a 5×5 neighborhood. Figure 4 and Figure 5 show chl-a outputs for the field campaign date 20 April 2007 prior to and after filtering, respectively. This image is affected by the presence of cirrus clouds and thus suboptimal atmospheric conditions. This leads to a high variation in the atmospheric correction output and consequently to high chl-a variations, which are removed by selective filtering. The images also show two regional limitations of the data processing: (1) narrow Lake Überlingen is frequently excluded from processing due to the influence of adjacency effects, and (2) Untersee results are often missing or reaching the algorithm threshold, due to large shallow water areas and possibly bad representation by the SIOP (specific inherent optical properties) optimized for Obersee.

### 3.2. Algorithm parameterization

**Figure 6.**
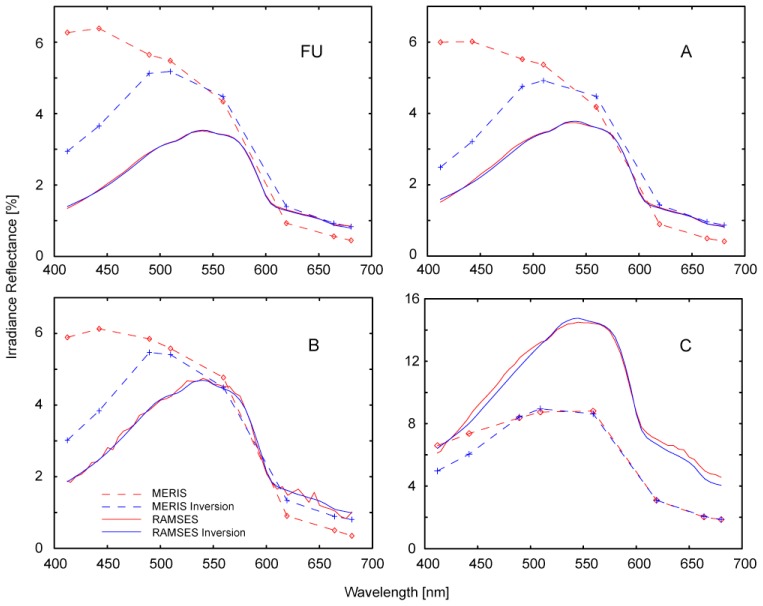
MERIS and RAMSES irradiance reflectance spectra for the sites FU and A-C (Figure 1) on 20 April 2007, with corresponding model spectra as resulting from inversion iterations. The concentrations calculated for inversion results are in Table 5.

MIP is originally used with input variables for individual optimization with sensors (i.e. channel weighting), aquatic regions (i.e. SIOP) or atmospheric conditions (i.e. aerosol models). For operational use, a lake-specific parameterization for best performance with all datasets is required, approximating the spatio-temporal variation of hydro-optic conditions. Iterative, image-based optimization is applied to determine aerosol model, AOT estimation channel and sm a priori assumption. Largest differences are found for different aerosol types, with continental aerosols leading to an underestimation of reflectances in short wavelength channels and finally to an overestimation of chl-a and low sm. Channel 14 (Table 1) measures in between the water vapor absorption bands, and has therefore performed best in the estimation of AOT with this algorithm [10]. The optimization of SIOP is done with the RAMSES measurements of 20 April 2007 and previous projects in Lake Constance [4, 24]. Measured R^-^ is inverted with absorption and scattering coefficients known from literature (Table 3). For b_b, sm_, we started iterations with a known exponential function [25], and adjusted the constants in factor and exponent, for a constant b_b_/b ratio of 0.0019 [4], which leads to a generally good agreement of modeled and measured RAMSES spectra (Figure 6). Reference spectra with high sm concentrations (i.e. Alpenrhein plume) are modeled less adequately than others, but an improved agreement for these sites can only be achieved by reducing the spectral exponent S [26] of y to 0.012 or by introducing an absorbing part of sm with S=0.012. The reason for this could be a significant portion of detritus absorption, which is not the case in other parts Lake Constance. In order not to decrease the model quality for the typical range of conditions, we neglected this change in SIOP. Iterations within certain thresholds are started with initial values (Table 4) unless values of adjacent pixels are available.

The chl-a concentrations of RAMSES and MERIS inversion and fluorometer measurements reveal an overestimation by RAMSES in sites A-C. In FU, the RAMSES inversion produced higher y absorption than in the other sites, but outputs a relatively low chl-a concentration. These two parameters can act as substitutes in the inversion and therefore cause certain discrepancies. Another uncertainty lies in the high spatio-temporal variation on the border of the plume in the center of the main basin, which is visible in Figure 1 and might have changed during the 3 hours of reference data acquisition. The sm concentrations agree better, with only the RAMSES estimate of site C revealing a larger offset. Y estimates by MERIS are impossible due to low reliability of the calculated R^-^ in channels 1 and 2, especially with difficult atmospheric conditions such as on 20 April 2007.

### 3.3. Inversion parameterization

Figure 6 displays a good agreement of RAMSES and MERIS in channels 5-8 for FU, A and B. Channels 1-4 are overestimated, possibly due to the thin cirrus clouds observed on that day, which are not accounted for in the atmospheric correction. The inversion algorithm enables individual weighting to account for systematic differences in the channels' reliability. In site C, AOT is overestimated because of significantly higher sm than assumed a priori. However, similar offsets occur also in most data with low sm, when using MERIS' original calibration. Empirical recalibration factors were thus applied to compensate for the bias found between calibrated radiances and model calculations. This adjustment was found necessary in previous work [22, personal communication], but only processing other sensors or lakes will reveal to what extent this is due to inaccuracy of model or calibration.

21 pairs of concurring chl-a measurements and MERIS images in 2003-2005 are used as training data. They are processed with varying weightings of channels 1-8 in the water constituent retrieval module, and with varying empirical recalibration factors for channel 1, 2, 3 (water constituents) and 14 (atmospheric correction). The optimization is started with channel 14, whose original radiance values lead to frequent overestimations of AOT, and thus to zero subsurface reflectance in channels 1, 2, 6-8. The datasets are processed in iterations with channel 14 lowered in intervals of 0.5%, which changes AOT only by few percent, but has a distinctive impact on short wavelength channel reflectances. Water constituent retrieval was performed for each AOT estimate, and chl-a outputs were compared to IGKB values. The best agreement was found for 0.97. Similar but multivariate optimization iterations are performed with the channels used by the water constituent module, using correlation coefficients as optimization measure. Channel 1 is excluded from the retrieval, since it displays random offsets from model results. Similar problems are encountered with channels 2 and 3, but reduction in weighting and individual recalibration leads to better results than their exclusion. The lowest R^-^ used is normally channel 8, which is thus the first to become zero when AOT is overestimated. Channel 8's weighting was therefore also slightly reduced. Table 6 is an overview of the weighting and recalibration values.

## 4. Results

### 4.1. Training of empirical recalibration

**Figure 7.**
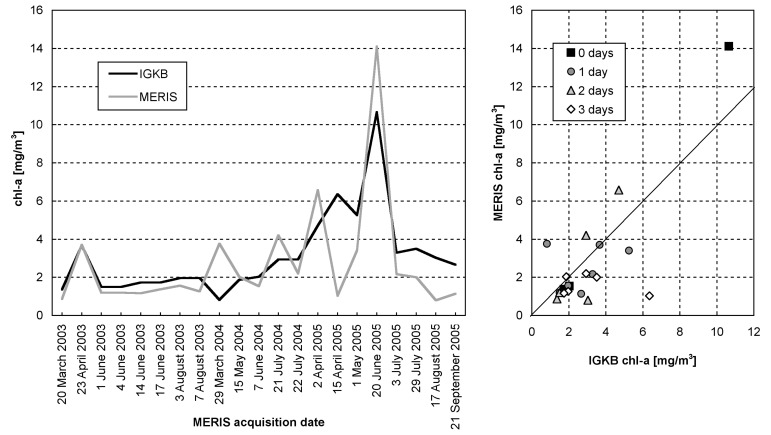
21 chl-a data pairs for the site FU, 2003-2005. The number of days between data acquisition are indicated in the figure on the right. MERIS values are filtered outputs, as shown in Figure 5.

**Figure 8.**
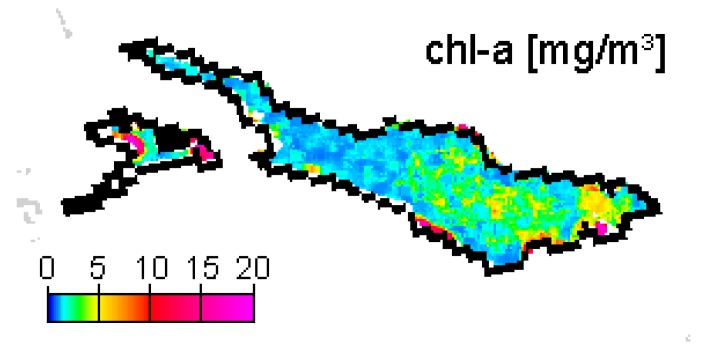
Chl-a concentration map for 15 April 2005.

The training data that were used in the recalibration reveal relatively low concentrations in 2003 and 2004, but high concentrations in 2005 (Figure 7). They contain data pairs for each spring bloom, but according to Lake Constance's natural variation, most data pairs represent chl-a concentrations between 1-4 mg/m^3^. The largest relative differences between satellite and sampling results are found for the datasets of 29 March 2004 and 15 April 2005. MERIS image of 29 March 2004 outputs high concentrations, while the corresponding IGKB measurement on 30 March 2004 is exceptionally low. However, a simultaneous probe profile reveals much higher values, and sample measurements acquired two weeks earlier and later confirm the spring bloom seen by MERIS. On 18 April 2005, IGKB measurements reach the spring bloom maximum of 6.4 mg/m^3^, while the corresponding estimate of MERIS for the sampling station FU on 15 April 2005 is remarkably low. However, MERIS derived concentrations are up to 5 mg/m^3^ in the eastern part of the main basin (Figure 8). A possible explanation could therefore be the spatio-temporal variation of algae, which can lead to significant differences for this data pair, where MERIS and IGKB acquisition lie 3 days apart (Figure 7, right). For the total 21 chl-a training data pairs, a correlation coefficient of 0.79 is achieved by iterative optimization of weighting and recalibration. If the images of 29 March 2004 and 15 April 2005 are excluded, the correlation coefficient increases to 0.94.

### 4.2. Validation

**Figure 9.**
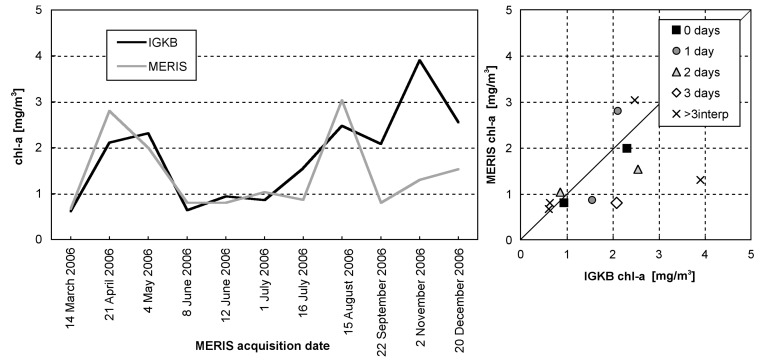
11 chl-a data pairs for validation of IGKB and MERIS measurements, for the site FU, 2006. Number of days between in situ sampling and satellite overpass are indicated in the figure on the right.

11 datasets acquired in 2006 were processed with the weighting and recalibration optimized for 2003-2005 data. The agreement with IGKB data is good for the first 8 datasets from March to August, correlating at a coefficient of 0.89, and representing the spring bloom, low chl-a in summer and an increase in August. However, an extraordinary increase in autumn is found in IGKB data, which is not found in MERIS imagery, leading to a low overall correlation (Figure 9).

**Figure 10.**
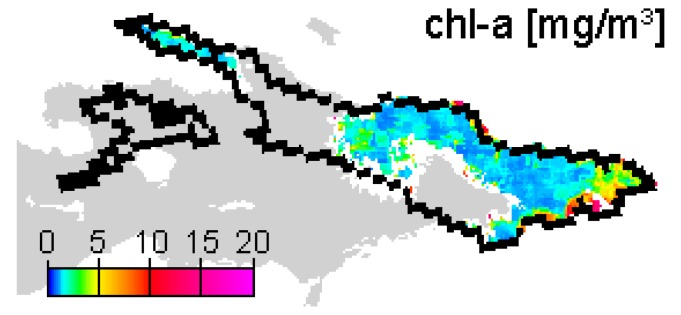
Chl-a concentration map for 22 September 2006. Grey color indicates bright pixel flags in MERIS data, white pixels within the shoreline are considered clouds by MIPs own masking algorithm.

**Figure 11.**
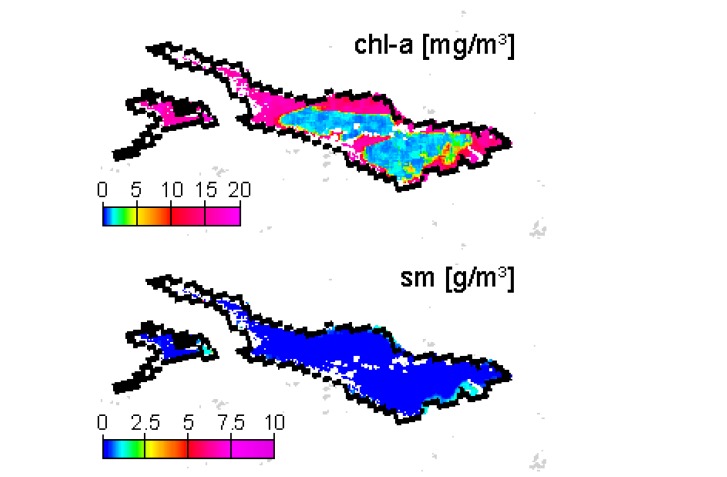
Chl-a and sm concentration maps for 2 November 2006. Pink and dark blue color represent threshold concentrations allowed by the algorithm, which indicates erroneous processing.

On 22 September 2006, 1-3 mg/m^3^ chl-a are calculated for the cloud free area around FU (Figure 10), thus a fairly good agreement with IGKB data. However, spatial variation is high, and the filtered FU geolocation pixel happens to output a significantly lower concentration value. The results for 2 November 2006 depict a more general explanation for the large differences in late 2006 data. The image based AOT retrieval is about 0.05 only, leading to inadequate atmospheric correction, and subsequently to erroneous water constituent output (Figure 11). In the IGKB measurements on 7 November 2006, Secchi depth of 8.3 m at FU is slightly above average, while chl-a samples reveal the maximum annual concentrations of 4.5 mg/m^3^ and 5.1 mg/m^3^ in the same week. However, high chl-a concentrations in the pelagic of Lake Constance normally lead to increased extinction and therefore low Secchi depth. A significant change in SIOP could therefore be a possible explanation for both the unexpected combination of high chl-a and high Secchi depth and the error in AOT estimation.

## 5. Conclusions and Discussion

This study confirms the general applicability of MIP for automatic, operational processing by applying a lake specific parameterization. The correlation of chl-a estimates from MERIS with in situ water quality monitoring is sufficient, considering differences in methodology and spatial representation. MERIS processing results are most reliable, when satellite estimates are validated by concurring in situ measurements, and applied for their additional spatial significance. Alternatively, MERIS chl-a results can be used as additional estimates, and thus improve the temporal resolution of current water quality monitoring. However, this approach requires the analysis of unvalidated MIP results, which are occasionally affected by processing errors. Expert knowledge is thus required in the interpretation of unvalidated outputs.

We distinguish three potential error sources introduced by the present processor, i.e. atmospheric correction, bio-optical parameterization and filtering. Atmospheric correction is the most fragile part. Errors in this module may be due to insufficient assumptions for atmospheric correction parameters (i.e. fixed aerosol model, sm a priori assumption). Adjacency effects are another source of atmospheric correction error and suspect of making most results of Lake Überlingen inadequate. Radiances in channel 14 thereby continuously increase towards the shore, leading to similarly increasing AOT estimates. This again leads to an underestimation of atmospherically corrected reflectances, especially in channels with high atmospheric scattering (1, 2) or low water reflectivity (6, 7, 8), where output reflectances can drop even to zero. The respective output concentrations are then either missing or equaling one of the threshold parameters, which are frequently found in areas within up to 5 pixels from the coastline [11]. Existing adjacency effect correction methods are currently considered for implementation [30, 31]. When large areas of the lake are unavailable in output, the reasons are either thin clouds ignored by MERIS quality flags or exceptionally high channel 14 radiances that cannot be accounted for with a constant sm backscattering assumption. A solution for the latter is the implementation of a more complex atmospheric correction module, which is iteratively coupled to the water constituent retrieval [22]. Neglecting MERIS' smile error could be another potential source of errors, although no camera border artifacts or correlation with observation zenith angle was found in the results.

The water constituent retrieval produces chl-a output that agrees well with FU sampling data, apart from the exceptional phytoplankton bloom in late 2006, where a change in SIOP seems to cause erroneous processing, with the simplified empirical parameterization being only a limited representation of the bio-optical complexity of the lake. However, the physical constitution enables arbitrary modifications to any single parameter where such problems occur, which could eventually lead to an alternative set of parameters to be specified for certain events that are known to lie out of range of the original parameterization.

The residual weighted filter improves the results significantly, by reducing aberrations by the algorithm due to atmospheric correction inaccuracies and at the same time performing spatial averaging to address the relatively low SNR in FR data. Moreover, the gap between spatially discrete laboratory samples and the complex representation of a spatially averaged, depth dependent estimate by remote sensing is hard to bridge. A conversion formula based on depth resolved profile measurements in Lake Überlingen [5] suggests that remote sensing generally underestimates sample measurements. This is not the case with our results, thus no conversion calculation was performed. However, the optimization of the channel recalibration with original IGKB data can be excluded as reason for this discrepancy, since it leads to large modifications in the processing of certain images, but not to a general scaling of the results.

An empirical recalibration of level 1B radiances was found necessary for the processing of MERIS data, with a majority of datasets producing erroneous or unreasonable output with the original calibration. The exact significance of this recalibration will only clarify with further investigation. It is expected that the processing chain can be applied to other large, prealpine lakes with the same recalibration, and individually optimized parameterizations only. Other than that, we consider the complementary use of and adjustment for MODIS data, whereof experience is available from previous work with MIP.

## Figures and Tables

**Table 1.** Operational MERIS band set [9].

**Band**	**Wavelength** **[nm]**	**Width** **[nm]**	**Potential Applications**

1	412.5	10	Yellow substance, turbidity
2	442.5	10	Chlorophyll absorption maximum
3	490	10	Chlorophyll, other pigments
4	510	10	Turbidity, suspended sediment, red tides
5	560	10	Chlorophyll reference, suspended sediment
6	620	10	Suspended sediment
7	665	10	Chlorophyll absorption
8	681.25	7.5	Chlorophyll fluorescence
9	705	10	Atmospheric correction, red edge
10	753.75	7.5	Oxygen absorption reference
11	760	2.5	Oxygen absorption R-branch
12	775	15	Aerosols, vegetation
13	865	20	Aerosols corrections over ocean
14	890	10	Water vapor absorption reference
15	900	10	Water vapor absorption, vegetation

**Table 2.** Overview of MERIS datasets used in this study.

**Year**	**Initial set**	**Sun glint**	**Cirrus or contrails**	**MIP error**	**Working set**	**Purpose**

2003	11	1	1	1	8	Training
2004	10	2	2	1	5	Training
2005	12	3	0	1	8	Training
2006	16	2	2	1	11	IGKB Validation
2007	2	0	1	0	1	Field validation
*Total*	*51*	*8*	*6*	*4*	*33*	

**Table 3.** Parameters used for analysis of Lake Constance (1).

**Process**	**Parameter**	**Value**
*Atmospheric Correction* (L_S_ to R_L_^-^)	Aerosol model	Maritime [10]
	AOT estimation	MERIS channel 14 [10]
	sm assumption	1.5 g/m^3^ [10]
*Water Constituent Retrieval* (R_L_^-^ to chl-a, sm, y)	a_w_	Buiteveld et al. [27]
	a_chl-a_	Heege [5]*0.75
	a_y_	S=0.014 [28]
	b_w_	Smith and Baker [29]
	b_b, sm_	0.014(λ/400)^n^ n=-0.8(λ/400)^1.2^ b_b_/b=0.019 [5]

**Table 4.** Parameters used for analysis of Lake Constance (2, values from [5]).

**Constituent**	**Initial value**	**Min. threshold**	**Max. threshold**
chl-a [mg/m^3^]	3	0.3	20
sm [g/m^3^]	1.5	0.2	10
y [m^-1^ (440 nm)]	0.2	0.1	0.35

**Table 5.** 20 April 2007 reference measurements (lab) sampled at 0.5 to 1 m depth, inversion results for RAMSES (ram, Figure 2) and MERIS (mer). MERIS acquisition time was at 9:46 UTC. MERIS pixel results are after filtering, results may thus vary slightly from the spectra in Figure 6.

**Site**	**UTC**	**Chl -a [mg/m^3^]**			**s m [g/m^3^]**			**y [m^-1^] (400 nm)**	
	**UTC ram**	**situ**	**ram**	**mer**	**situ**	**ram**	**mer**	**ram**	**mer**
FU	8:20	0.8	1.1	1.4	0.6	0.6	0.8	0.25	0.11
A	9:25	1.1	1.9	1.1	0.8	0.7	0.7	0.21	0.10
B	10:20	1.1	1.3	0.9	1.0	0.9	0.7	0.22	0.10
C	11:05	3.6	4.9	3.2	2.3	3.9	1.7	0.20	0.12

**Table 6.** Weighting and recalibration factors for MERIS bands 1-8 and 14 (Table 1), which were used for water constituents and AOT retrieval, respectively.

**Channel**	**1**	**2**	**3**	**4**	**5**	**6**	**7**	**8**	**14**

Recalibration	-	0.975	0.98	-	-	-	-	-	0.97
Weighting	-	0.2	0.5	1	1	1	1	0.8	0.97
